# Risk factors for metachronous colorectal cancer and advanced neoplasia following primary colorectal cancer: a systematic review and meta-analysis

**DOI:** 10.1186/s12876-023-03053-2

**Published:** 2023-11-30

**Authors:** Ye Zhang, Amalia Karahalios, Ye Kyaw Aung, Aung Ko Win, Alex Boussioutas, Mark A. Jenkins

**Affiliations:** 1 Center for Epidemiology and Biostatistics, Melbourne School of Population and Global Health, University of Melbourne, Parkville, Victoria 3010 Australia; 2 University of Melbourne Centre for Cancer Research, Victorian Comprehensive Cancer Centre, University of Melbourne, Parkville, Victoria 3010 Australia; 3 Genetic Medicine, Royal Melbourne Hospital, Parkville, Victoria 3050 Australia; 4 Department of Medicine, Royal Melbourne Hospital, University of Melbourne, Parkville, Victoria 3050 Australia; 5 Department of Gastroenterology, The Alfred, Monash University, Melbourne, Victoria 3800 Australia; 6 Department of Medicine, Central Clinical School, Monash University, Clayton, Australia

**Keywords:** Colorectal cancer, Metachronous, Risk factors, Systematic review

## Abstract

**Background:**

Identifying risk factors for metachronous colorectal cancer (CRC) and metachronous advanced neoplasia could be useful for guiding surveillance. We conducted a systematic review and meta-analysis to investigate risk factors for metachronous CRC and advanced neoplasia.

**Methods:**

Searches were conducted in MEDLINE, Embase, Web of Science and Cochrane Central Registry of Controlled Trials for articles (searching period: 1945 to Feburary, 2021) that reported the results of an association between any factor and metachronous advanced neoplasia or metachronous CRC. There were no restrictions on the publication date or language. Random effects models were fitted to estimate the combined association between the risk factors and metachronous CRC or advanced neoplasia. The Risk of Bias In Non-Randomised Studies of Interventions tool (ROBINS-I) was used to assess the risk of bias of included studies.

**Results:**

In total, 22 observational studies with 625,208 participants were included in the systematic review and meta-analysis. Of these, 13 studies investigated risk factors for metachronous CRC and 9 for advanced neoplasia. The risks of metachronous CRC or advanced neoplasia were higher if the first CRC was diagnosed in the presence of a synchronous advanced lesion (pooled risk ratio (RR) from 3 studies: 3.61, 95% confidence interval (CI): 1.44–9.05; and pooled RR from 8 studies: 2.77, 95% CI: 2.23–3.43, respectively). The risk of metachronous CRC was lower, but the risk of metachronous advanced neoplasia was higher if the first CRC was distal (compared with proximal) (pooled RR from 3 studies: 0.48, 95% CI: 0.23–0.98; and pooled RR from 2 studies: 2.99, 95% CI: 1.60–5.58 respectively). The risk of metachronous advanced neoplasia increased with age (pooled RR from 3 studies: 1.07 per year of age, 95% CI: 1.03–1.11). There was no evidence that any lifestyle risk factors studied were associated with the risk of metachronous CRC or advanced neoplasia.

**Conclusions:**

The identified risk factors for metachronous CRC and advanced neoplasia might be useful to tailor the existing surveillance guidelines after the first CRC. There were potential limitations due to possible misclassification of the outcome, confounding and risk of bias, and the findings cannot be generalised to high-risk genetic syndrome cases.

**Supplementary Information:**

The online version contains supplementary material available at 10.1186/s12876-023-03053-2.

## Introduction

Colorectal cancer (CRC) is the third most commonly diagnosed cancer in the world [1]. The majority of CRCs develop via the adenoma pathway [2]. Aging populations and the increasing prevalence of lifestyle risk factors are increasing the incidence of CRC. Decreasing CRC mortality (particularly in high-income countries [1]) due to improved treatments, and increased early detection via screening for CRC, are increasing the number of CRC survivors and length of survival time. In combination, these conditions are increasing the number of people at risk of metachronous CRC (i.e., a new primary CRC following an initial CRC) or advanced adenoma in the remaining colorectum [3]. The reported risk of metachronous CRC within 5 years after curative surgical resection of the colon and rectum ranges from 2 to 12 % [4, 5].

Following treatment of the first CRC, surveillance colonoscopy is currently recommended for detecting metachronous CRC and metachronous advanced adenoma. However, current surveillance guidelines [6, 7] are the same for all CRC survivors (i.e., a one-size-fits-all approach) because the risk factors for metachronous CRC and advanced adenoma are poorly understood.

Previous reviews [6–8] have found that those whose first CRC had high-grade dysplasia and tubulovillous architecture and had a synchronous CRC or adenoma, were more likely to be diagnosed with a metachronous CRC. However, these reviews differed by study population and definition of metachronous CRC. For example, Gupta et al. summarised risk factors for metachronous CRC among individuals whose first event was an adenoma (not a CRC ) [7]. Jayasekara et al. summarized risk factors for both metachronous adenoma and metachronous CRC among individuals whose first event was either an adenoma or CRC [8]. Kahi et al. provided a narrative review of the risk factors for metachronous CRC after resection for the initial CRC without conducting a meta-analysis [6]. In this systematic review, we used meta-analysis to separately quantify the effect for the pre-specified risk factors (i.e., exposures), and two metachronous events (i.e., metachronous CRC and metachronous advanced neoplasia (for studies that did not distinguish between advanced adenoma or CRC events)).

## Methods

We reported a systematic review using the Preferred Reporting Items for Systematic Review and Meta-analyses-2020 (PRISMA-2020, [9] (Supplementary Material 1). Methods were predetermined and registered on the International Prospective Register of Systematic Reviews (PROSPERO ID: CRD42021237512).

### Eligibility criteria

We defined metachronous CRC as a new primary CRC diagnosed at least 6 months after the first CRC [3]. For articles that combined metachronous CRC with advanced adenoma (i.e., did not present results separately for metachronous CRC and adenoma), we defined metachronous advanced neoplasia as a diagnosis of any of the following conditions that occurred at least 6 months after the first CRC: tubular adenoma ≥10 mm in diameter; adenoma with villous or tubulovillous histology; adenoma with high-grade dysplasia; or primary CRC [10]. We planned to include articles that reported the results of randomised controlled trials (RCTs) or observational studies that reported hazard ratios, odds ratios, risk ratios, standardized incidence or rate ratios (and corresponding 95% confidence intervals (CIs)) as the association between any risk factor and metachronous advanced neoplasia or metachronous CRC. We reported on those aged 18 years or older when diagnosed with the first CRC. We excluded articles where the metachronous and synchronous CRC could not be differentiated; or the minimal interval between the first CRC and second CRC or metachronous advanced neoplasia was undefined or was less than 6 months; or metachronous CRC or advanced neoplasia included anastomotic or local/regional recurrence; or only included participants at high risk of metachronous CRC (e.g., Lynch syndrome); or was unpublished; or only published as a letter to the editor, conference abstract, editorial, review of articles, or a commentary. We did not apply any language restrictions.

### Searches

Searches were conducted on 15th February 2021 in MEDLINE (Ovid), Embase (Ovid), Cochrane Central Registry of Controlled Trials and Web of Science. The searching period was set from 1945 to February 2021. An updated search was conducted on 25th July 2022. Search strategies were developed with a biomedical librarian using the following terms: (“metachronous” AND (“colorectal cancer” OR “colon cancer” OR “rectal cancer” or “bowel cancer”) AND “risk factors”).

### Screening

The full search strategies are provided in Supplementary Material 2. All screening was undertaken independently by two authors (YZ and YKA). First, all titles and abstracts of the articles were independently assessed against the eligibility criteria. Next, the full text of the articles that appeared to meet the eligibility criteria was assessed against the eligibility criteria. Finally, references of eligible papers and a review article [8] were checked for additional eligible articles not identified by the initial search.

### Data extraction

For studies that met the eligibility criteria, the following data were independently extracted by two authors (YZ and YKA): study characteristics (title, first author’s last name, year of publication, location of the study, study design, sample size, follow-up time); participant characteristics (age, sex); CRC related characteristics (definition of metachronous CRC or advanced neoplasia); risk factors assessed, and factors that were adjusted for in multivariable analyses. Disagreements were resolved by a third author (MAJ). Where data were not provided in the published manuscript, two authors (as a joint email from YZ and MAJ) contacted the corresponding authors by email (with one reminder) seeking unpublished data.

### Risk of bias assessment

Observational studies meeting the eligibility criteria were critically appraised for risk of bias independently by two authors (YZ and YKA) according to the Risk of Bias in Non-Randomised Studies of Interventions tool (ROBINS-I) [11]. The following six domains were included in the assessment: bias due to confounding, bias in the selection of participants into the study, bias in classification of interventions, bias due to missing data, bias in measurement of outcomes and bias in the selection of reported result. For the assessment of bias due to confounding, we used directed acyclic graphs to decide a priori that the following factors would be considered as potential confounders of any association with metachronous CRC or advanced neoplasia: age, sex, country, education, alcohol, smoking, physical activity, dietary factors, body mass index, stage of first tumour, grade of first tumour, site of first tumour, resection treatment, family history of CRC, polyp history, screening. We planned to use the Risk of Bias 2 tool [12] to assess the risk of bias of randomised trials but no trials were identified that met our eligibility criteria.

### Statistical analysis

Associations that were reported in single studies could not be pooled and were presented as reported. Where multiple studies were available, hazard ratios and risk ratios were combined as a measure of pooled risk ratio (RR) given they estimate similar effects since the outcomes are rare [13]. For each study, the extent of association that was extracted was the one that was maximally adjusted (i.e., adjusted for the covariates). Given the diversity of the included studies and to generalize the meta-analytic results beyond the included studies, random-effects models using the restricted maximum likelihood (REML) estimator for the between-study variance (*τ*^2^) were fitted to estimate the pooled RRs and corresponding 95% confidence intervals (CIs). The 95% CIs were calculated using the Knapp-Hartung method when more than four studies were available [14]. Forest plots were used to display the pooled RRs and 95% CI for the associations between each risk factor and outcome. The I^2^ statistic was used to assess heterogeneity across selected studies. Results were reported separately for metachronous CRC and metachronous advanced neoplasia. We planned to use funnel plots to assess small study effects if more than 10 studies were included in the meta-analysis for each risk factor. We conducted sensitivity analyses where we excluded studies that did not adjust for age and/or sex because age and sex are both considered to be important confounding variables. We also conducted a *post-hoc* sensitivity analysis for the association between age and metachronous CRC to exclude one study [15] that reported a risk ratio in the opposite direction to those reported by the other studies. To address the effect of time period on the clinical evolution (e.g., endoscopy instruments, techniques), we first sorted studies by study year (i.e., midpoint of the study period) to visually assess if there appeared to be a trend by time. If an obvious trend was presented, we planned to fit meta-regression models by including study year as a continuous variable. Any amendments from the protocol are provided in Supplementary Material 3.

The associations between age at diagnosis of the first CRC and metachronous CRC, and advanced neoplasia were reported for various categorisations of age: continuous e.g., per year of age; binary e.g., ≥60 years vs. < 60 years; or nominal e.g., decades of age. To combine these, we converted the effect estimates for the reported associations to correspond to ‘per year of age’ and pooled them using a random effects model [16]. To convert the effect estimate for studies reporting categories of age, we needed a single value for each category of age. Where the category was bounded, we assigned the age value as the midpoint of the age category. If the highest age category was open-ended, we assigned the age value to be the value of the lower bound plus the width of the interval of the previous (second-to-highest) category. If the lowest age category was open-ended, we assigned the age value to be the value of the upper bound minus the width of the next interval [17, 18]. We excluded studies that did not report the number of participants as the method required this information.

All statistical analyses were performed using Stata version 17.0 (Stata Corp LP, College Station, TX, USA ) [19].

## Results

**Fig. 1:**
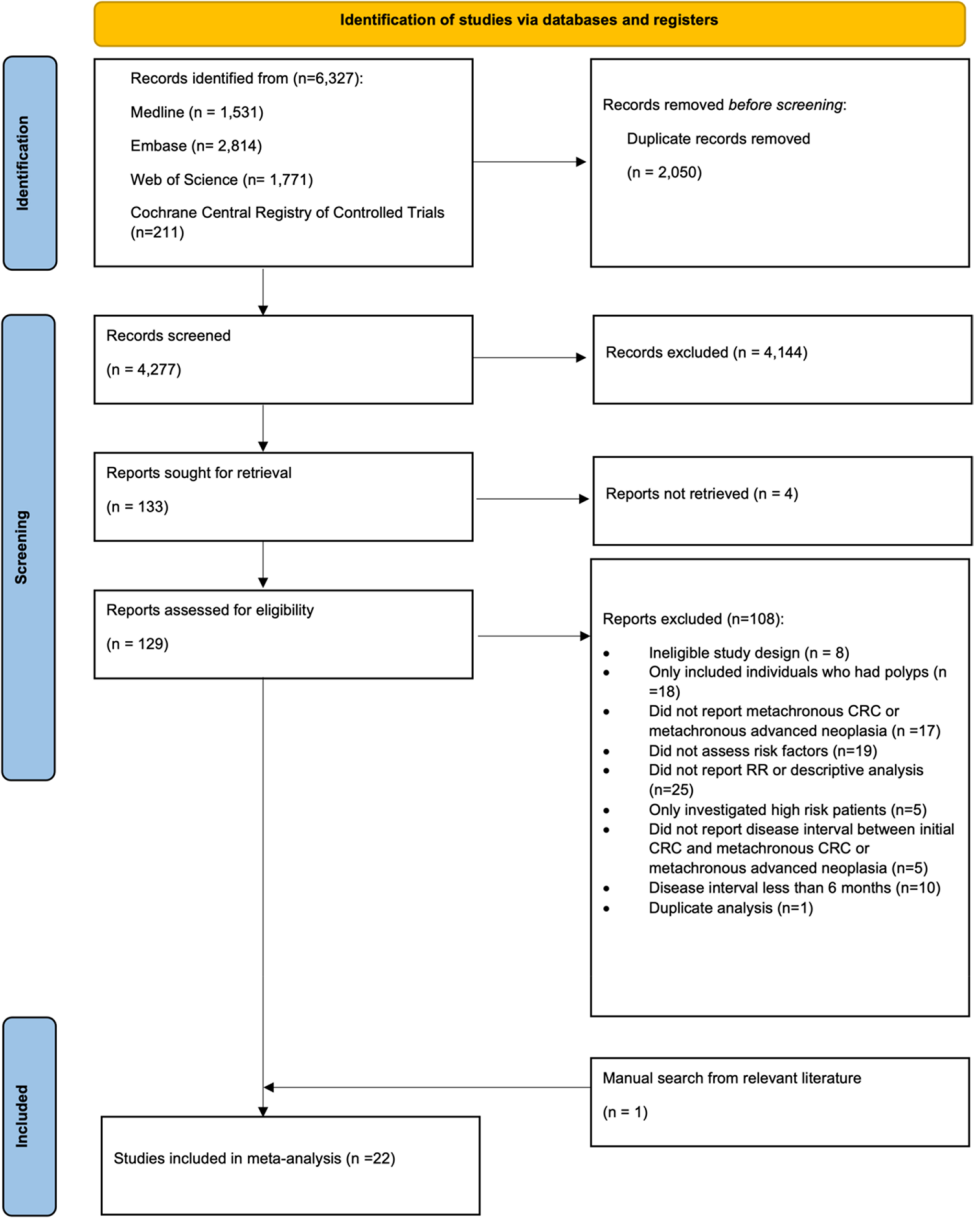
Flow diagram for study selection for risk factors for metachronous colorectal cancer and advanced neoplasia

Our search identified 6327 studies. After removing duplicates, 4277 titles and abstracts were screened for eligibility. Of these, 133 appeared to meet the eligibility criteria, 4 studies were unable to be retrieved, and 129 studies were assessed for eligibility using the full text. Of these, 108 studies were excluded leaving 21 studies included in this review. An additional study was found through a manual search from the references of the eligible studies. In total, 22 articles of 625,208 participants were included in this systematic review and meta-analysis (Fig. 1).

Of the included studies, 13 investigated risk factors for metachronous CRC [4, 15, 20–30], and 9 for metachronous advanced neoplasia [31–39]. Characteristics of included studies are shown in Tables 1 and 2. All 22 studies were cohort studies. Seven studies were conducted in Europe [4, 22–24, 28, 36, 38], ten studies in Asia [15, 27, 29–32, 34, 35, 37, 39], four studies in USA [20, 25, 26, 33] and one across multiple countries [21].

**Table 1:** Characteristics of included study of risk factors for metachronous colorectal cancer

First author’s last name [ref]	Year	Country	Study design	Total cohort population	Age at diagnosis	Sex	Follow-up time	Definition of outcome	Assessed risk factors	Confounders adjusted for in the analysis
Yang [20]	2018	USA	Retrospective Cohort	252,404	Range < 40–80+	128,396 M + 124008F	0 - > 8 years	CRC with a minimal interval of 6 months between the diagnoses was required to exclude synchronous cancer.	History of initial primary CRC, age at diagnosis, sex, race, SEER staging, if surgery, pathology grade	History of initial primary CRC, age at diagnosis, sex, race, SEER staging, if surgery, pathology grade
Jayasekara [21]	2016	USA, Australia, Canada, New Zealand	Retrospective Cohort	7863	Mean 55.3 years	3969 M + 3894F	Mean 6.6 years (range 1–16 years)	A metachronous CRC was defined as a new primary colon or rectal cancer diagnosed at least one year after the first diagnosis of primary colon or rectal cancer.	Age, sex, family history, synchronous CRC, synchronous adenoma, site, stage, grade, mismatch repair status, smoking, alcohol, body mass index, diabetes mellitus, aspirin, ibuprofen, multivitamin use, calcium intake, surveillance colonoscopy interval	Age, sex, family history, synchronous CRC, synchronous adenoma, site, stage, grade, mismatch repair status, smoking, alcohol, body mass index, diabetes mellitus, aspirin, ibuprofen, multivitamin use, calcium intake, surveillance colonoscopy interval
le Clercq [22]	2015	Netherlands	Retrospective Cohort	5157	Mean 70.0 years	2770 M + 2387F	Not given	A second primary colorectal adenocarcinoma diagnosed at least 6 months after the primary CRC diagnosis, and which was not a recurrence of the primary CRC.	Age, tumour size, appearance, differentiation	Age and sex
Battersby [23]	2014	UK	Prospective Cohort	538	Median 70 years 11 months (IQR, 61 years 10 months–77 years 11 months)	295 M + 243F	Median 4 years and 2 months (range 0–16 years)	Metachronous colorectal cancers are tumours that develop at a site remote from the primary tumour, which are histologically separate and occur 12 months or more after surgery, in a completely investigated colon.	Age	Competing events (death and recurrence), sex, stage, site, and number of polyps at initial colonoscopy
Mulder [24]	2012	Netherlands	Retrospective Cohort	10,283	median 70.0 years	5199 M + 5084F	39,974 person-years	Pathologically proven CRC away from the previous line of anastomosis at least 6 months after the first diagnosis	Age, sex, TNM stage, synchronous CRC, time interval in between first and second diagnoses	Rate ratio of standardized incidence ratios standardized for age, sex, TNM stage, time interval in between first and second diagnoses
Raj [25]	2011	USA	Retrospective Cohort	104,257	65.6 years ±10.9 (SD) for colon cancer and 62.8 years ±11.5 (SD) for rectal cancer	56,705 M + 47552F	618,104 person-years	Second CRC was diagnosed more than 6 months of the first diagnosis	Sex, ethnicity	Adjusted for age
Park [15]	2006	Korea	Retrospective Cohort	5447	Mean 55 years	3208 M + 2239F	Not given	A metachronous cancer was defined as a second primary colorectal cancer occurring more than 6 months after the index cancer without evidence of local recurrence or metastasis from the primary tumour	Age (≤40 years), sex, family history of CRC and related cancer, location of index cancer, differentiation, existence of synchronous polyps or cancer	Age, sex, family history of CRC and related cancer, location of index cancer, differentiation, existence of synchronous polyps or cancer
Das [26]	2006	USA	Retrospective Cohort	216,751	50 years or more	M + F	1,250,687 person years	Either in situ or malignant and microscopically confirmed and diagnosed subsequent to the index CRC more than 6 months	Age, sex, site, stage of first primary, time period of diagnosis of first primary (1973–1977 vs 1988–1992)	Age, sex, site, stage of first primary, time period of diagnosis of first primary (1973–1977 vs 1988–1992)
Yamazaki [27]	1997	Japan	Retrospective Cohort	284	Mean 59.5 years	174 M + 110F	Mean 53 months	Neoplastic findings after the first six-month surveillance	Age, sex, tumour stage, tumour site, tumour grade, depth of invasion, lymph node metastasis, carcinoma in other organs, familial history of colorectal carcinoma, synchronous adenoma or carcinoma	Age, sex, tumour stage, tumour site, tumour grade, depth of invasion, lymph node metastasis, carcinoma in other organs, familial history of colorectal carcinoma, synchronous adenoma or carcinoma
Bouvier [4]	2008	France	Retrospective Cohort	10,801	Median 71.1 years	5998 M + 4803F	61,879 person years	A new colorectal cancer occurring at least 6 months after the initial colorectal cancer	Sex, size, growth features, stage of first cancer, synchronous CRC, associated adenomas or adenoma remnants	Sex, size, growth features, stage of first cancer, synchronous CRC, associated adenomas or adenoma remnants
Gervaz [28]	2005	Switzerland	Retrospective Cohort	5006	Range 20–85+	M + F	Not given	A pathologically proved adenocarcinoma that distinctly separated from the previous line of anastomosis and diagnosed at a minimal interval of 6 months after the initial carcinoma. The possibility that the second lesion represents a recurrence must be ruled out beyond reasonable doubt	Location of the index cancer	Not mentioned
Shitoh [29]	2002	Japan	Retrospective Cohort	272	Not given	M + F	Median 75 months	CRC more than 1 year after the first primary CRC	Coexistent adenoma, family history of CRC, past history of extracolonic malignancy (all malignant neoplasms were included), MSI status	Coexistent adenoma, family history of CRC, past history of extracolonic malignancy (all malignant neoplasms were included), MSI status
Togashi [30]	2000	Japan	Retrospective Cohort	341	Mean 59.6 years	209 M + 132F	Mean 6.2 years	All CRCs detected in surveillance colonoscopy which occurred more than 6 months after the surgery	Concurrent adenoma, synchronous CRC, extracolonic malignancy, family history of CRC in first degree relative	Concurrent adenoma; synchronous CRC; extracolonic malignancy; family history of CRC in first degree relative

**Table 2:** Characteristics of included study of risk factors for metachronous advanced neoplasia

First author’s last name [ref]	Year	Country	Study design	Total cohort population	Age at diagnosis	Sex	Follow-up time	Definition of outcome	Assessed risk factors	Confounders adjusted for in the analysis
Minamide [31]	2021	Japan	Retrospective Cohort	343	Median 65 years (IQR: 59–71)	219 M+ 124F	Median 61.5 months (IQR: 41.4–66.2)	Metachronous advanced neoplasia defined as adenoma ≥10 mm, adenoma with villous histology, adenoma with high-grade dysplasia, or invasive cancer detected at least 6 months after pre-resection colonoscopy for SM-CRC	Age (≥65 yrs), sex, lesion location, resection method, synchronous advanced neoplasia, number of surveillance total colonoscopy	Age, sex, location (colon or rectum), resection method (SR or ER), and number of surveillance colonoscopies (< 3 or ≥ 3 times)
Nam [32]	2020	Korea	Retrospective Cohort	293	Mean 63.2 ± 10.4 years (range 34 to 89)	179 M + 114F	Mean 74.4 ± 36.4 months (range 12.3 to 185.1)	Colon neoplasms detected ≥1 year after perioperative clearing colonoscopy were defined as metachronous lesions	Age (≥65 yrs), sex, BMI, diabetes, hypertension, current alcohol drinking, current smoking, T stage, N stage, advanced stage (stage3), location, differentiation, synchronous high-risk adenoma	Age, sex, BMI, diabetes, hypertension, current alcohol drinking, current smoking, T stage, N stage, advanced stage (stage3, location, differentiation, synchronous high-risk adenoma
Tjaden [33]	2019	USA	Retrospective Cohort	697	less than 75	M + F	Not given	CRC or adenoma ≥10 mm in endoscopic size, villous histology or high-grade dysplasia or the presence of AN or ≥ 3 adenomas more than 6 months after the diagnosis of CRC	Age(< 50 yrs), sex, race, smoking status, aspirin usage, first-degree family history, BMI, cancer location, cancer side, tumour differentiation, cancer stage, synchronous adenoma, synchronous AN, side of synchronous AN, synchronous HRA, ≥3 synchronous adenomas, synchronous sessile serrated polyp, prepared quality at index	Adjusted for centre
Choe [34]	2015	Korea	Retrospective Cohort	451	Mean 61.47 ± 9.91 years	287 M + 164F	Median 48.7 months (range 12.1–108.0 month)	Metachronous CRC was defined as a second primary CRC occurring more than 1 year after the resection of the index cancer and does not include an anastomosis line recurrence or a locoregional recurrence invading the colorectum. An advanced adenoma was defined as an adenoma ≥10 mm in size, with a villous histology or high-grade dysplasia.	Age (< 65 vs. > 65 years), sex, the location of the index tumour (proximal to splenic flexure was right), obesity, diabetes, hypertension, aspirin use, hypercholesterolemia, alcohol consumption, cigarette smoking, stage, high risk patients	Age, sex, the location of the index tumour (proximal to splenic flexure was right), obesity, diabetes, hypertension, aspirin use, hypercholesterolemia, alcohol consumption, cigarette smoking, stage, high risk patients
Lee [35]	2014	Korea	Retrospective Cohort	1049	Mean 59.0 ± 10.3 years (range 26–85)	647 M + 402F	Mean 40.7 ± 19.1 months (range 7–75)	Metachronous neoplasms were defined as neoplasms and advanced adenoma occurring more than 6 months after the resection of primary neoplasm. They had to be distinctly separated by at least 4 cm from the anastomosis	Age, synchronous adenoma, diabetes mellitus, sex, hypertension, baseline multiple adenomas, synchronous advanced adenoma	Age, synchronous adenoma, diabetes mellitus, gender, hypertension, baseline multiple adenomas, synchronous advanced adenoma
Borda [36]	2012	Spain	Retrospective Cohort	382	Not given	M + F	Median 48 months	Metachronous lesion was defined as the lesion that appeared at least 12 months after resection and was not located in the surgical anastomosis	40 variables on patient characteristics, initial neoplastic lesions, and immunohistochemical CRC features	40 variables on patient characteristics, initial neoplastic lesions, and immunohistochemical CRC features
Moon [37]	2010	Korea	Retrospective Cohort	503	Mean 58.6 years	328 M + 175F	Mean 35.7 months, range 6 to 84 months	A lesion arising from mucosa at a site other than anastomosis found in a surveillance colonoscopy more than 6 months after resection	Age, sex, stratification of synchronous neoplasia, time of first follow-up colonoscopy	Age, sex, stratification of synchronous neoplasia, time of first follow-up colonoscopy
Balleste [38]	2007	Spain	Retrospective Cohort	355	Mean 67 years	223 M + 132F	Up to 2 years	Any adenoma or carcinoma arising from mucosa at a site other than anastomosis between the first and second year of follow-up	Sex, undifferentiated tumour, previous or synchronous adenoma, previous CRC	Sex, undifferentiated tumour, previous CRC
Yabuuchi [39]	2018	Japan	Retrospective Cohort	1731	Median 66 years (IQR 59–72)	1075 M + 656F	Median 47.5 months (IQR 24.4–48.4)	A new CRC or advanced adenoma diagnosed at least more than 12 months after the initial CRC	Age (≥65 years), sex, surgical resection type, synchronous advanced neoplasia, TNM stage, family history of colorectal cancer, diabetes mellitus, adjuvant chemotherapy, body mass index, aspirin use, alcohol, smoking	Age (≥65 years), sex, surgical resection type, synchronous advanced neoplasia, TNM stage, family history of colorectal cancer, diabetes mellitus, adjuvant chemotherapy, body mass index, aspirin use, alcohol, smoking

### Risk factors for metachronous CRC

**Fig. 2:**
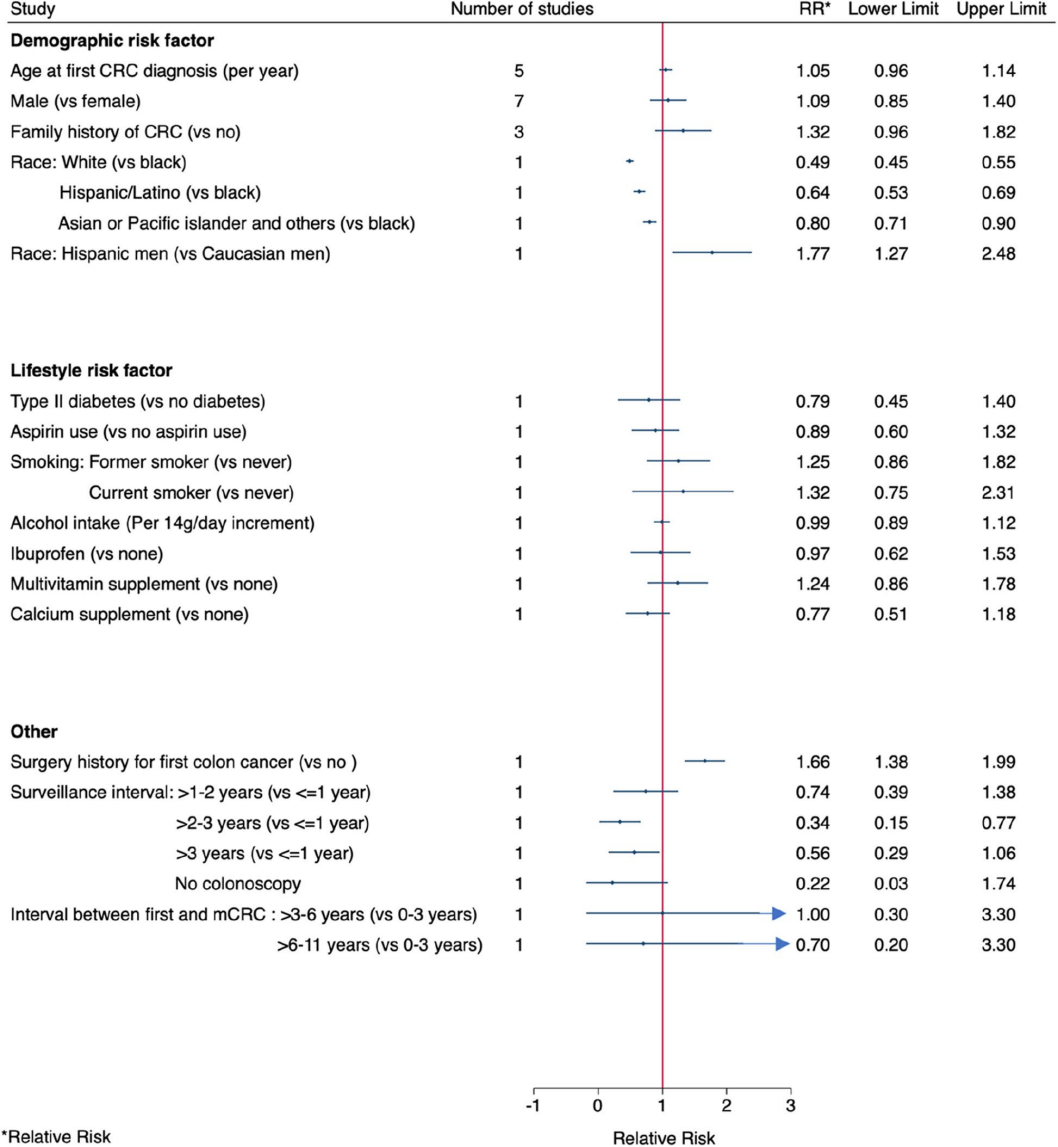
Pooled or single-study association estimates between demographic, lifestyle, and other factors and metachronous colorectal cancer

There was no evidence of an association with age (5 studies, pooled RR per year = 1.05, 95% CI: 0.96–1.14, I^2^ = 96%, Fig. 2 and Fig. S1), sex (7 studies, pooled RR for male vs female =1.09, 95% CI: 0.85–1.40, I^2^ = 82%, Fig. 2 and Fig. S2), or first-degree family history of CRC (compared to no family history) (3 studies, pooled RR = 1.32, 95% CI, 0.96–1.82, I^2^ = 0%, Fig. 2 and Fig. S3) and metachronous CRC.

Of the 22 eligible studies, only one reported lifestyle factors (type II diabetes, aspirin use, smoking status, alcohol intake, ibuprofen use, multivitamin supplementation, and calcium supplementation )[21], and none were found to be associated with metachronous CRC (Fig. 2).

**Fig. 3:**
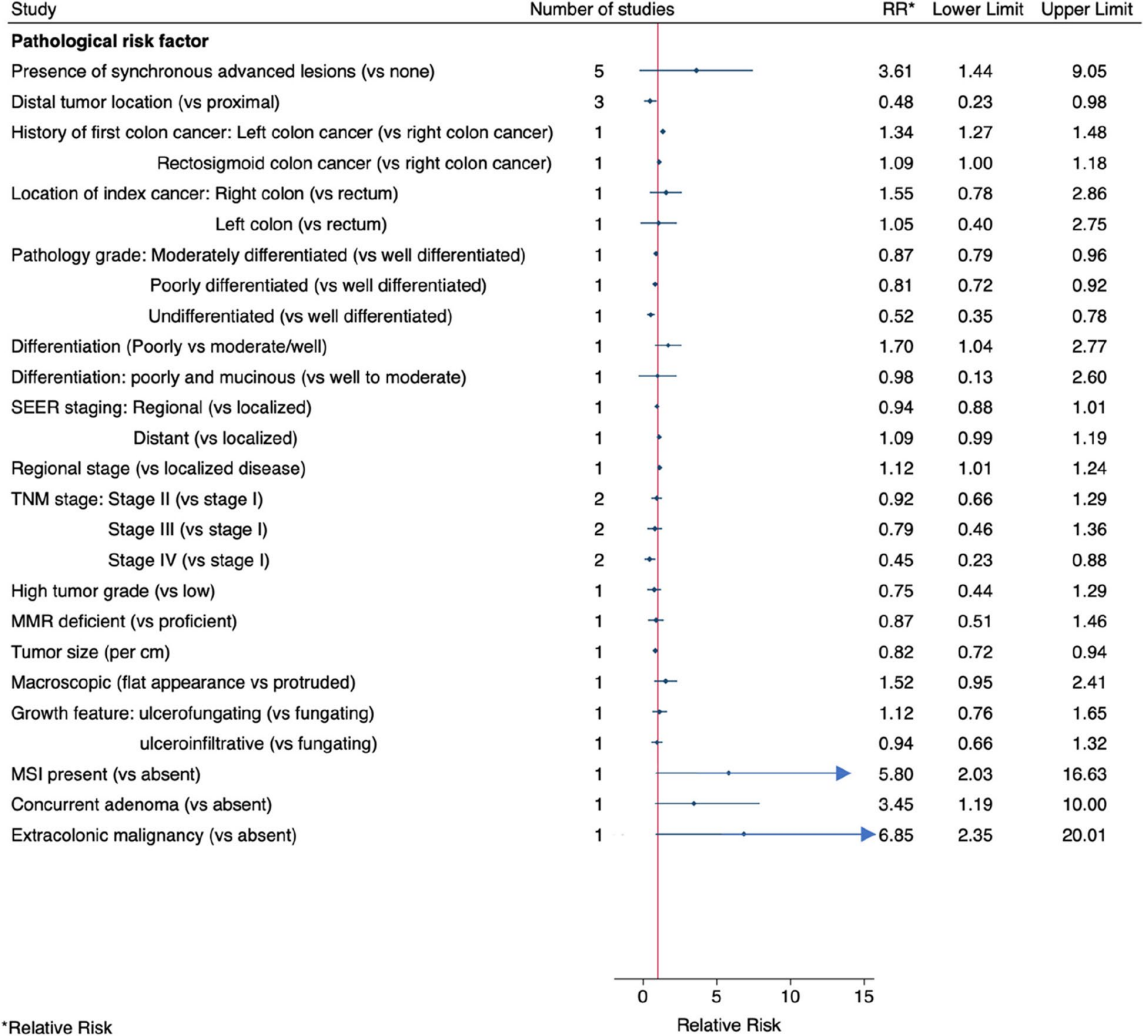
Pooled or single-study estimates for the association between pathological factors and metachronous colorectal cancer

Having a synchronous lesion was associated with a 3.6-fold increased risk of metachronous CRC with relatively high heterogeneity (5 studies, pooled RR = 3.61, 95% CI: 1.44–9.05, I^2^ = 66%, Fig. 3 and Fig. S9). A distal CRC (compared to a proximal CRC) was associated with decreased risk of metachronous CRC with high heterogeneity (3 studies, pooled RR = 0.48, 95% CI: 0.23–0.98, I^2^ = 94%, Fig. 3 and Fig. S10).

### Risk factors for metachronous advanced neoplasia

**Fig. 4:**
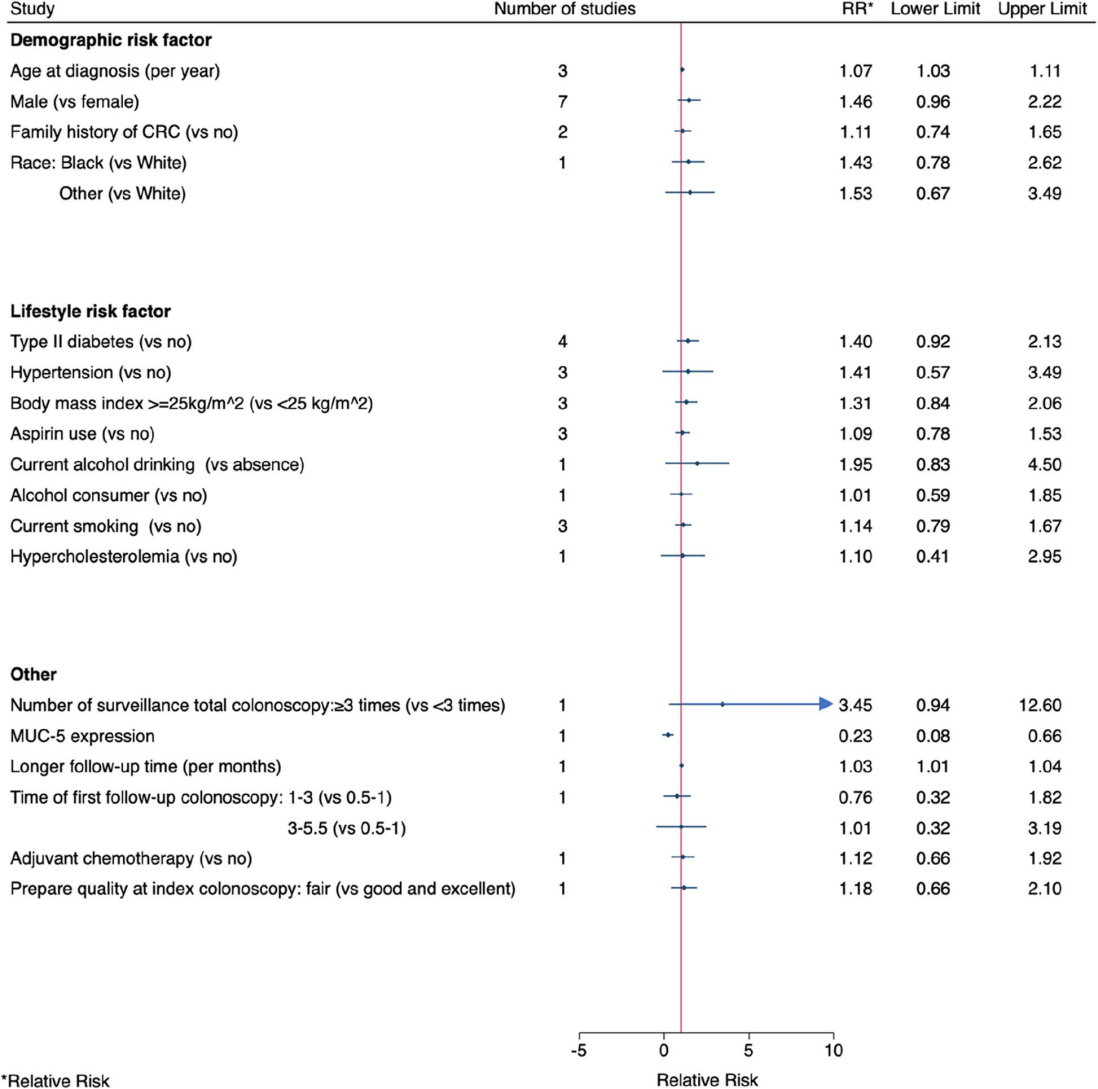
Pooled or single-study association estimates between demographic, lifestyle, and other factors and metachronous advanced neoplasia

Nine studies investigated demographic risk factors for metachronous advanced neoplasia. Advancing age was associated with an increased risk of developing metachronous advanced neoplasia with no heterogeneity (3 studies, pooled RR per year =1.07, 95% CI: 1.03–1.11, I^2^ = 0%, Fig. 4 and Fig. S1). There was no evidence of an association with sex with moderate heterogeneity (7 studies, pooled RR for males vs females =1.46, 95% CI: 0.96–2.22, I^2^ = 38%, Fig. 4 and Fig. S2). There was no evidence of an association with family history of CRC and no observed heterogeneity between the studies (2 studies, pooled RR = 1.11, 95% CI: 0.74–1.65, I^2^ = 0%, Fig. 4 and Fig. S3).

There was no evidence of an association with type 2 diabetes (4 studies, pooled RR = 1.40, 95% CI: 0.92–2.13, I^2^ = 0%, Fig. 4 and Fig. S4), hypertension (3 studies, pooled RR = 1.41, 95% CI: 0.57–3.49, I^2^ = 9%, Fig. 4 and Fig. S5), being overweight, (3 studies, pooled RR = 1.31, 95% CI: 0.84–2.06, I^2^ = 0%, Fig. 4 and Fig. S6); aspirin use (3 studies, pooled RR = 1.09, 95% CI: 0.78–1.53, I^2^ = 0%, Fig. 4 and Fig. S7), or current smoking (3 studies, pooled RR = 1.14, 95% CI: 0.79–1.67, I^2^ = 0%, Fig. 4 and Fig. S8).

**Fig. 5:**
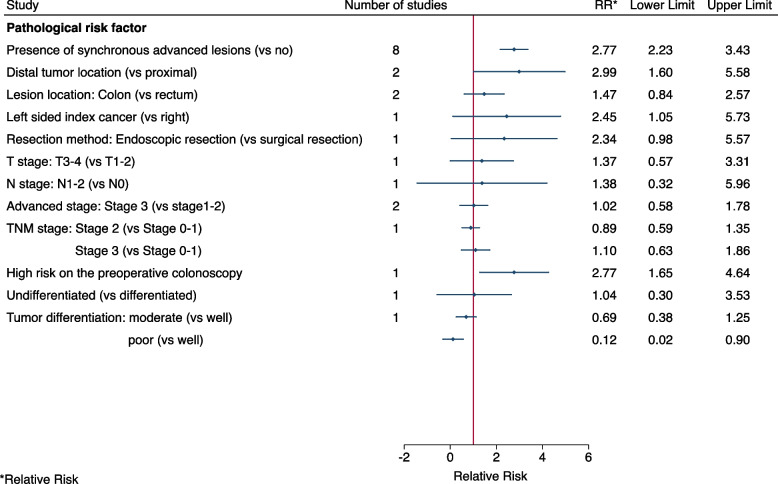
Pooled or single-study estimates of the association between pathological factors and metachronous advanced neoplasia

Having a synchronous lesion was associated with a 3-fold increased risk and no heterogeneity was observed (8 studies, pooled RR = 2.77, 95% CI: 2.23–3.43, I^2^ = 0%, Fig. 5 and Fig. S9). The first CRC being distal, was associated with a three-fold increased risk (2 studies, pooled RR = 2.99, 95% CI: 1.60–5.58, I^2^ = 1%, Fig. 5 and Fig. S10). There was no evidence of an association with advanced stage (stage III) of the first CRC, compared with stages I-II and no heterogeneity was observed (2 studies, pooled RR = 1.02, 95% CI: 0.58–1.78, I^2^ = 0%, Fig. 5 and Fig. S11).

### Sensitivity analysis, publication bias and meta-regression

After excluding studies that did not adjust for age or sex, there were no material changes in the pooled estimates of associations for sex, family history of CRC or stage with the development of metachronous CRC, or in the association between synchronous advanced lesions and metachronous advanced adenoma, or the association between age and metachronous CRC. Removing the study that reported a risk ratio in the opposite direction to those reported by the other studies in our sensitivity analyses did not materially change the pooled estimate of the age association. We did not conduct an assessment of small study effects because none of our pooled estimates were based on more than 10 studies. Only one risk factor (i.e., presence of advanced lesions) is likely to be affected by improvements in clinical practice (e.g. instrument improvement). For this risk factor, we included eight studies and when we ordered the studies by the midpoint of the study period, we found no indication that the effect changed with time; therefore, we did not conduct the meta-regression.

### Risk of bias assessment

None of the articles included all of the important confounders that we determined a priori. Accordingly, we assessed most articles as having a moderate to serious risk of bias in the confounder domain. All but one study [27] was classified as having a low risk of bias in the selection of participants into the study (the one study had serious risk of bias in this domain). All but one study [27] was classified as having a low risk of bias in the classification of interventions (again, the one study had serious risk of bias). All studies had a moderate risk of bias due to missing data given there were missing data for various covariates. All studies were classified as having low to moderate risk of bias in measurement of outcomes, and selection of reported results. Overall, most articles had moderate risk of bias except for three articles that had a serious risk of bias (Table S1).

## Discussion

In this systematic review, we reviewed 22 articles to summarize the evidence of risk factors and the outcomes, metachronous CRC and advanced neoplasia. We confirmed the findings of previous systematic reviews that the risks of metachronous CRC and advanced neoplasia are 3–4 fold greater if the first primary CRC had a synchronous advanced lesion. We also found that those with a first CRC in the distal colon (compared with the proximal colon) were at increased risk of metachronous advanced neoplasia but lower risk of metachronous CRC. None of the other risk factors (demographic or lifestyle) we investigated appeared to be associated with metachronous CRC or advanced neoplasia.

### Strengths and limitations

We employed a comprehensive search strategy of four databases to maximise the identification of eligible studies, resulting in a larger number of studies. We also conducted a dose-response analysis for age at diagnosis of first CRC to include as many reported associations; this has not been done previously. There are some potential limitations to our study. We limited our analysis to articles that defined metachronous as neoplasia diagnosed at least 6 months after the primary and metachronous CRC. While this definition reduces misclassification of synchronous tumours as metachronous, it potentially excludes studies of true metachronous CRC diagnosed within 6 months. We also excluded studies that focused on high-risk individuals, including those with a genetic syndrome, and therefore we cannot generalise the findings to such patients. As all studies were observational (not randomised as the risk factors we assessed cannot be randomised), they all had a potential to be biased, at least in one of the domains assessed by the risk of bias assessment. Although most studies attempted to adjust for potential important confounders, we cannot rule out residual confounding that might have biased the effect estimates. To minimise this issue, we restricted our analysis to those that at least minimally adjusted for age and sex.

### Comparison to other studies

The association of age with metachronous CRC or advanced neoplasia remains inconclusive. Our results differ from previous meta-analyses [8] as our analytic method allowed us to include all studies that assessed age as a risk factor, whether they treated age as a categorical or continuous risk factor. We found evidence for an increased risk of 3–11% (RR per year =1.07, 95% CI: 1.03–1.11) of advanced neoplasia for each year older than the first CRC was diagnosed. This could be due to an ageing bowel being more susceptible to a second cancer, but we cannot rule out a simpler explanation that an ageing bowel is at increased risk of any cancer. In contrast, Park et al. [15] found that being younger than age 40 years at first CRC diagnosis was associated with an increased risk of metachronous CRC by 6-fold (RR: 6.37, 95% CI: 2.51–16.15) compared to people older than 40 years. This study might have estimated a different effect compared with the other studies because it had a cohort of younger participants resulting in a longer follow-up time. Removing this study from our sensitivity analysis did not materially change the pooled estimate of the age association.

Consistent with a previous systematic review [8], we also observed that having synchronous advanced lesions increased the risk of developing both metachronous CRC and advanced neoplasia. Guidelines recommend that patients with synchronous advanced lesions at their initial surgery be followed intensely by colonoscopy. We found a lower risk of metachronous CRC, but a higher risk of metachronous advanced neoplasia for those with a distally located initial CRC. One potential explanation might be that it takes longer for a CRC to form. Usually, this is through advanced adenoma, which are detected and removed by surveillance thereby altering the natural history of CRC formation.

We found no evidence that lifestyle risk factors contributed to the risk of metachronous CRC or advanced neoplasia. However, it is difficult to rule out such associations given most of the studies in our review collected information about lifestyle risk factors before the primary CRC, when ideally the exposure would occur since the primary CRC diagnosis. A diagnosis of CRC could result in changes to lifestyle and behavior, for example quitting smoking and drinking less alcohol [40]. We also found insufficient evidence for the association between diabetes and metachronous CRC. However, a real association could have been missed due to length-time bias masking any associations, given CRC cases with diabetes may be more likely to die due to diabetic complications before developing metachronous CRC. We observed marginal evidence that being overweight was associated with increased risk of metachronous advanced neoplasia but not metachronous CRC. Many of the risk factors were only assessed in a single study and therefore were not amenable to meta-analyses, for example lifestyle risk factors for metachronous CRC (e.g., smoking, alcohol intake, multivitamin and calcium supplementation), pathological risk factors (e.g., tumour differentiation and size) and surveillance interval. Therefore, compared to the more commonly studied risk factors, power to observe associations was low.

Existing guidelines [41] have clear expectations on the surveillance of patients after curative resection, albeit they are based on low levels of evidence. Recommendations are not nuanced with consideration of risk factors. About 50% of metachronous CRC occur within 2 years after the primary CRC and hence the first colonoscopy after CRC treatment is critical for patients. Australian guidelines published by Cancer Council Australia and endorsed by the National Health and Medical Research Council (last updated in 2017) recommend a colonoscopy 1 year after curative primary resection and then, after a clear colonoscopy, a further colonoscopy every 5 years [42]. This approach is a one-size-fits-all which could be more targeted if risk factors could be identified, including this systematic review and meta-analysis. For example, more intensive screening could be recommended for those with a synchronous CRC or polyps at the time of diagnosis of first CRC.

## Conclusions

In conclusion, we comprehensively reviewed the current evidence for a series of risk factors for metachronous CRC and metachronous advanced neoplasia and conducted meta-analyses to obtain pooled estimates. Similar to other studies, we found that synchronous advanced lesions and the location of initial CRC (proximal vs distal) were associated with the risk of developing metachronous CRC and metachronous advanced neoplasia, and we did not identify any additional risk factors. Existing studies were generally small with short follow-up periods. Future studies aiming to identify new risk factors will need to be larger, i.e., thousands of cases of colorectal cancer followed prospectively over 10 years and have detailed information on a wide range of risk factors.

### Supplementary Information

**Additional file 1.** Supplementary material 1: PRISMA checklist.

**Additional file 2.** Supplementary material 2: Full search strategies for the systematic review of the literature on risk factors for metachronous colorectal cancer and advanced neoplasia.

**Additional file 3.** Supplementary material 3: Summaries of amendments from registered protocol.

**Additional file 4: Figure S1.** Forest plot of the association between age and metachronous advanced neoplasia and metachronous colorectal cancer. (a) including all selected studies (b) sensitivity analysis of excluding one study reported the opposite association. All relative risks (RRs) were calculated through dose-response analysis. CI, confidence interval. **Figure S2.** Forest plot of the association between sex and metachronous advanced neoplasia and metachronous colorectal cancer. (a) including all selected studies (b) sensitivity analysis of excluding unadjusted effect estimates. RR, relative risk; CI, confidence interval. **Figure S3.** Forest plot of the association between family history and metachronous advanced neoplasia and metachronous colorectal cancer. (a) including all selected studies (b) sensitivity analysis of excluding unadjusted effect estimates. RR, relative risk; CI, confidence interval. **Figure S4.** Forest plot presenting meta-analysis for the association between type 2 diabetes (vs no) and metachronous advanced neoplasia and metachronous colorectal cancer. RR, relative risk; CI, confidence interval. **Figure S5.** Forest plot presenting meta-analysis for the association between hypertension (vs no) and metachronous advanced neoplasia. RR, relative risk; CI, confidence interval. **Figure S6.** Forest plot presenting meta-analysis for the association between body mass index *(> = 25 kg/m*^*2*^ vs *< 25 kg/m*^*2*^*)* and metachronous advanced neoplasia. RR, relative risk; CI, confidence interval. **Figure S7.** Forest plot presenting meta-analysis for the association between aspirin use and metachronous advanced neoplasia and metachronous colorectal cancer. (a) including all selected studies (b) sensitivity analysis of excluding unadjusted effect estimates. RR, relative risk; CI, confidence interval. **Figure S8.** Forest plot presenting meta-analysis for the association between current smoking and metachronous advanced neoplasia. RR, relative risk; CI, confidence interval. **Figure S9.** Forest plot presenting meta-analysis for the association between presence of synchronous advanced lesions and metachronous advanced neoplasia and metachronous colorectal cancer. (a) including all selected studies (b) sensitivity analysis of excluding unadjusted effect estimates. RR, relative risk; CI, confidence interval. **Figure S10.** Forest plot presenting meta-analysis for the association between location of first colorectal cancer (distal vs proximal) and metachronous advanced neoplasia and metachronous colorectal cancer. (a) including all selected studies (b) sensitivity analysis of excluding unadjusted effect estimates. RR, relative risk; CI, confidence interval. **Figure S11.** Forest plot presenting meta-analysis for the association between advanced TNM stage and metachronous advanced neoplasia. RR, relative risk; CI, confidence interval.

**Additional file 5: Table S1.** Risk of bias assessment summary by Risk of Bias in Non-Randomized Studies of “Interventions” (ROBINS-I) tool.

## Data Availability

All data generated or analysed during this study are included in this published article.

## Abbreviations

CRC: Colorectal Cancer
RCT: Randomised Control Trial
ROBINS-I: Risk of Bias in Non-Randomized Studies of “Interventions”
RR: Risk Ratio
CI: Confidence Interval
REML: Restricted Maximum Likelihood
