# Supplementary material for: Risk factors for metachronous colorectal cancer and advanced neoplasia following primary colorectal cancer: a systematic review and meta-analysis
Source: BMC Gastroenterol. 2023 Nov 30;23:421. doi: 10.1186/s12876-023-03053-2 (PMC10688466; doi:10.1186/s12876-023-03053-2)
Supplement: Supplementary file 2 — Additional file 2. Supplementary material 2: Full search strategies for the systematic review of the literature on risk factors for metachronous colorectal cancer and advanced neoplasia. [file 12876_2023_3053_MOESM2_ESM.docx]

**Supplementary material 2. Full search strategies for the systematic review of the literature on risk factors for metachronous colorectal cancer and advanced neoplasia.**

Last search: July 2022

**Medline (Ovid) search strategy**

1. exp colorectal neoplasms/ or adenomatous polyposis coli/ or colonic neoplasms/ or colorectal neoplasms, hereditary nonpolyposis/ or rectal neoplasms/

2. ((colorectal or rectal or bowel or colon) adj5 (cancer or neoplasm* or neoplasia or carcinoma)).mp. [mp=title, abstract, original title, name of substance word, subject heading word, floating sub-heading word, keyword heading word, organism supplementary concept word, protocol supplementary concept word, rare disease supplementary concept word, unique identifier, synonyms]

3. 1or 2

4. Neoplasms, Second Primary/

5. (second adj3 colorectal).mp. [mp=title, abstract, original title, name of substance word, subject heading word, floating sub-heading word, keyword heading word, organism supplementary concept word, protocol supplementary concept word, rare disease supplementary concept word, unique identifier, synonyms]

6. Metachronous.mp. [mp=title, abstract, original title, name of substance word, subject heading word, floating sub-heading word, keyword heading word, organism supplementary concept word, protocol supplementary concept word, rare disease supplementary concept word, unique identifier, synonyms]

7. 4 or 5 or 6

8. risk*.mp. [mp=title, abstract, original title, name of substance word, subject heading word, floating sub-heading word, keyword heading word, organism supplementary concept word, protocol supplementary concept word, rare disease supplementary concept word, unique identifier, synonyms]

9. risk/ or risk factors/

10. incidence/

11. incidence.mp. [mp=title, abstract, original title, name of substance word, subject heading word, floating sub-heading word, keyword heading word, organism supplementary concept word, protocol supplementary concept word, rare disease supplementary concept word, unique identifier, synonyms]

12. hazard ratio.mp.

13. 8 or 9 or 10 or 11 or 12

14. 3 and 7 and 13

**The same search strategy as in Medline (Ovid) was used in EBM-Cochrane Central Register of Controlled Trials.**

**Embase (Ovid) search strategy**

1. exp colorectal tumor/ or exp colon tumor/ or exp rectum tumor/ or exp colorectal adenoma

2. ((colorectal or rectal or bowel or colon) adj5 (cancer or neoplasm* or neoplasia or carcinoma)).mp. [mp=title, abstract, heading word, drug trade name, original title, device manufacturer, drug manufacturer, device trade name, keyword, floating subheading word, candidate term word]

3. 1 or 2

4. exp second cancer/

5. (second adj3 colorectal).mp. [mp=title, abstract, heading word, drug trade name, original title, device manufacturer, drug manufacturer, device trade name, keyword, floating subheading word, candidate term word]

6. Metachronous. mp. [mp=title, abstract, heading word, drug trade name, original title, device manufacturer, drug manufacturer, device trade name, keyword, floating subheading word, candidate term word]

7. 4 or 5 or 6

8. risk*.mp. [mp=title, abstract, heading word, drug trade name, original title, device manufacturer, drug manufacturer, device trade name, keyword, floating subheading word, candidate term word]

9. incidence.mp. [mp=title, abstract, heading word, drug trade name, original title, device manufacturer, drug manufacturer, device trade name, keyword, floating subheading word, candidate term word]

10. hazard ratio.mp. [mp=title, abstract, heading word, drug trade name, original title, device manufacturer, drug manufacturer, device trade name, keyword, floating subheading word, candidate term word]

11. 8 or 9 or 10

12. 3 and 7 and 11

**Web of science search strategy**

#1 TS=(colorectal neoplasms or adenomatous polyposis coli or colonic neoplasms or colorectal neoplasms, hereditary nonpolyposis or rectal neoplasms)

#2 TI=(colorectal NEAR/5 cancer or neoplasm* or neoplasia or carcinoma)

#3 TI=(colon NEAR/5 cancer or neoplasm* or neoplasia or carcinoma)

#4 TI=(rectal NEAR/5 cancer or neoplasm* or neoplasia or carcinoma)

#5 TI=(bowel NEAR/5 cancer or neoplasm* or neoplasia or carcinoma)

#6 #5 OR #4 OR #3 OR #2 OR #1

#7 TS=“second primary”

#8 TS=Metachronous

#9 TI=(second NEAR/3 colorectal)

#10 #9 OR #8 OR #7

#11 TS=(risk* or risk factor* or incidence or hazard ratio*)

#12 #11 AND #10 AND #6
