# Supplementary material for: Risk factors for metachronous colorectal cancer and advanced neoplasia following primary colorectal cancer: a systematic review and meta-analysis
Source: BMC Gastroenterol. 2023 Nov 30;23:421. doi: 10.1186/s12876-023-03053-2 (PMC10688466; doi:10.1186/s12876-023-03053-2)
Supplement: Supplementary file 3 — Additional file 3. Supplementary material 3: Summaries of amendments from registered protocol. [file 12876_2023_3053_MOESM3_ESM.docx]

**Supplementary Material 3 Summaries of amendments from registered protocol**

| **Protocol method** | **Deviation from protocol method, with justification** |
| --- | --- |
| 1. We planned simply treated age as a binary variable to analyse the association between age of diagnosis of initial colorectal cancer (CRC) and metachronous CRC or metachronous advanced neoplasia. 2. We did not mention any sensitivity analysis in the protocol. | 1. We conducted a dose response analysis for age at diagnosis to include all reported associations between age and metachronous CRC or metachronous advanced neoplasia.   *Types of deviation: addition*   1. We conducted sensitivity analysis where excluding studies did not adjust for age and/or sex.   We conducted sensitivity analysis for age associations to exclude one study that reported a risk ratio that was in the opposite direction to those by other studies.  *Types of deviation: addition* |
