# Supplementary material for: Risk factors for metachronous colorectal cancer and advanced neoplasia following primary colorectal cancer: a systematic review and meta-analysis
Source: BMC Gastroenterol. 2023 Nov 30;23:421. doi: 10.1186/s12876-023-03053-2 (PMC10688466; doi:10.1186/s12876-023-03053-2)
Supplement: Supplementary file 4 — Additional file 4: Figure S1. Forest plot of the association between age and metachronous advanced neoplasia and metachronous colorectal cancer. (a) including all selected studies (b) sensitivity analysis of excluding one study reported the opposite association. All relative risks (RRs) were calculated through dose-response analysis. CI, confidence interval. Figure S2. Forest plot of the association between sex and metachronous advanced neoplasia and metachronous colorectal cancer. (a) including all selected studies (b) sensitivity analysis of excluding unadjusted effect estimates. RR, relative risk; CI, confidence interval. Figure S3. Forest plot of the association between family history and metachronous advanced neoplasia and metachronous colorectal cancer. (a) including all selected studies (b) sensitivity analysis of excluding unadjusted effect estimates. RR, relative risk; CI, confidence interval. Figure S4. Forest plot presenting meta-analysis for the association between type 2 diabetes (vs no) and metachronous advanced neoplasia and metachronous colorectal cancer. RR, relative risk; CI, confidence interval. Figure S5. Forest plot presenting meta-analysis for the association between hypertension (vs no) and metachronous advanced neoplasia. RR, relative risk; CI, confidence interval. Figure S6. Forest plot presenting meta-analysis for the association between body mass index (> = 25 kg/m2 vs < 25 kg/m2) and metachronous advanced neoplasia. RR, relative risk; CI, confidence interval. Figure S7. Forest plot presenting meta-analysis for the association between aspirin use and metachronous advanced neoplasia and metachronous colorectal cancer. (a) including all selected studies (b) sensitivity analysis of excluding unadjusted effect estimates. RR, relative risk; CI, confidence interval. Figure S8. Forest plot presenting meta-analysis for the association between current smoking and metachronous advanced neoplasia. RR, relative risk; CI, confidence interval. Figure S9 [file 12876_2023_3053_MOESM4_ESM.docx]

**Figure S1. Forest plot of the association between age and metachronous advanced neoplasia and metachronous colorectal cancer. (a) including all selected studies (b) sensitivity analysis of excluding one study reported the opposite association. All relative risks (RRs) were calculated through dose-response analysis. CI, confidence interval.**

(a)

(b)

**Figure S2. Forest plot of the association between sex and metachronous advanced neoplasia and metachronous colorectal cancer. (a) including all selected studies (b) sensitivity analysis of excluding unadjusted effect estimates. RR, relative risk; CI, confidence interval.**

(a)

*Male vs Female

(b)

*Male vs Female (excluding unadjusted effect estimates)

**Figure S3. Forest plot of the association between family history and metachronous advanced neoplasia and metachronous colorectal cancer. (a) including all selected studies (b) sensitivity analysis of excluding unadjusted effect estimates. RR, relative risk; CI, confidence interval.**

(a)

*First degree family history of CRC

(b)

*First degree family history of CRC (excluding unadjusted effect estimates)

**Figure S4. Forest plot presenting meta-analysis for the association between type 2 diabetes (vs no) and metachronous advanced neoplasia and metachronous colorectal cancer. RR, relative risk; CI, confidence interval.**

*Type II diabetes

**Figure S5. Forest plot presenting meta-analysis for the association between hypertension (vs no) and metachronous advanced neoplasia. RR, relative risk; CI, confidence interval.**

**Figure S6. Forest plot presenting meta-analysis for the association between body mass index *(>=25kg/m^2^ vs <25kg/m^2^)* and metachronous advanced neoplasia. RR, relative risk; CI, confidence interval.**

* BMI (BMI>=25kg/m^2^ vs <25kg/m^2^)

**Figure S7. Forest plot presenting meta-analysis for the association between aspirin use and metachronous advanced neoplasia and metachronous colorectal cancer. (a) including all selected studies (b) sensitivity analysis of excluding unadjusted effect estimates. RR, relative risk; CI, confidence interval.**

(a)

*Ever use aspirin vs never use

(b)

*Ever use aspirin vs never use (excluding unadjusted effect estimates)

**Figure S8. Forest plot presenting meta-analysis for the association between current smoking and metachronous advanced neoplasia. RR, relative risk; CI, confidence interval.**

*Current smoking (vs non-smoking)

**Figure S9. Forest plot presenting meta-analysis for the association between presence of synchronous advanced lesions and metachronous advanced neoplasia and metachronous colorectal cancer. (a) including all selected studies (b) sensitivity analysis of excluding unadjusted effect estimates. RR, relative risk; CI, confidence interval.**

(a)

(b)

*Excluding unadjusted effect estimates

**Figure S10. Forest plot presenting meta-analysis for the association between location of first colorectal cancer (distal vs proximal) and metachronous advanced neoplasia and metachronous colorectal cancer. (a) including all selected studies (b) sensitivity analysis of excluding unadjusted effect estimates. RR, relative risk; CI, confidence interval.**

(a)

*Distal vs proximal

(b)

*Distal vs proximal (excluding unadjusted effect estimates)

**Figure S11. Forest plot presenting meta-analysis for the association between advanced TNM stage and metachronous advanced neoplasia. RR, relative risk; CI, confidence interval.**

*Advanced TNM stage (vs stage I/II) defined as TNM stage III
