# Supplementary material for: Risk factors for metachronous colorectal cancer and advanced neoplasia following primary colorectal cancer: a systematic review and meta-analysis
Source: BMC Gastroenterol. 2023 Nov 30;23:421. doi: 10.1186/s12876-023-03053-2 (PMC10688466; doi:10.1186/s12876-023-03053-2)
Supplement: Supplementary file 5 — Additional file 5: Table S1. Risk of bias assessment summary by Risk of Bias in Non-Randomized Studies of “Interventions” (ROBINS-I) tool. [file 12876_2023_3053_MOESM5_ESM.docx]

**Table S1. Risk of bias assessment summary by Risk of Bias in Non-Randomized Studies of Interventions (ROBINS-I) tool.**

|  | **Bias due to confounding** | **Bias in selection of participants into the study** | **Bias in classification of interventions** | **Bias due to missing data** | **Bias in measurement of outcomes** | **Bias in selection of the reported result** | **Overall bias** |
| --- | --- | --- | --- | --- | --- | --- | --- |
| Yang, 2018 | Moderate | Low | Low | Moderate | Moderate | Low | Moderate |
| Jayasekara, 2016 | Moderate | Low | Low | Moderate | Low | Low | Moderate |
| le Clercq, 2015 | Moderate | Low | Low | Moderate | Low | Moderate | Moderate |
| Battersby, 2014 | Moderate | Low | Low | Moderate | Low | Low | Moderate |
| Mulder, 2015 | Moderate | Low | Low | Moderate | Low | Low | Moderate |
| Raj, 2011 | Serious | Low | Low | Moderate | Low | Low | Serious |
| Park, 2006 | Moderate | Low | Low | Moderate | Low | Low | Moderate |
| Das, 2006 | Moderate | Low | Low | Moderate | Low | Low | Moderate |
| Yamazaki, 1997 | Moderate | Serious | Serious | Moderate | Moderate | Moderate | Serious |
| Bouvier, 2008 | Moderate | Low | Low | Moderate | Low | Low | Moderate |
| Gervaz, 2005 | Serious | Low | Low | Moderate | Low | Low | Serious |
| Shitoh, 2002 | Moderate | Low | Low | Moderate | Low | Low | Moderate |
| Togashi, 2000 | Moderate | Low | Low | Moderate | Low | Low | Moderate |
| Minamide, 2021 | Moderate | Low | Low | Moderate | Low | Low | Moderate |
| Nam, 2020 | Moderate | Low | Low | Moderate | Moderate | Low | Moderate |
| Tjaden, 2019 | Moderate | Low | Low | Moderate | Low | Low | Moderate |
| Choe, 2015 | Moderate | Low | Low | Moderate | Low | Low | Moderate |
| Lee, 2014 | Moderate | Low | Low | Moderate | Moderate | Low | Moderate |
| Borda, 2012 | Moderate | Low | Low | Moderate | Low | Low | Moderate |
| Moon, 2010 | Moderate | Low | Low | Moderate | Low | Low | Moderate |
| Balleste, 2007 | Moderate | Low | Low | Moderate | Low | Low | Moderate |
| Yabuuchi, 2018 | Moderate | Low | Low | Moderate | Low | Low | Moderate |
